# Dielectric and Magnetoelectric Properties of TGS–Magnetite Composite

**DOI:** 10.3390/molecules29061378

**Published:** 2024-03-20

**Authors:** Mariusz Trybus, Levan Chotorlishvili, Elżbieta Jartych

**Affiliations:** 1 Department of Physics and Medical Engineering, Rzeszow University of Technology, al. Powstancow Warszawy 12, 35-959 Rzeszow, Poland; m_trybus@prz.edu.pl (M.T.); l.chotorlish@prz.edu.pl (L.C.); 2 Department of Electronics and Information Technology, Faculty of Electrical Engineering and Computer Science, Lublin University of Technology, 20-618 Lublin, Poland

**Keywords:** triglycine sulphate, TGS, magnetite, composite material, dielectric properties, multiferroic

## Abstract

In our studies, we combined two powdered materials, i.e., ferroelectric triglycine sulfate (TGS) and ferrimagnetic magnetite Fe_3_O_4_, to obtain a magnetoelectric composite. The ferroelectric (E) part, i.e., TGS, was a hybrid organic–inorganic crystal, which we obtained as a pure single crystal from an aqueous solution using a static water evaporation method. The magnetic (M) part of the composite was commercially available magnetite. The samples used for the dielectric and magnetoelectric measurements were cold-pressed and made in the form of a circular tablet. The measuring electrodes were made of silver-based conductive paste and were attached to the sample. We measured the temperature dependencies of selected electrical parameters (e.g., dielectric permittivity, electrical capacity, and loss angle tangent). We used the dynamic lock-in method to check whether magnetoelectric coupling existed between the E and M phases. In this paper, we present the dielectric properties of pure monocrystalline TGS as a reference sample and compare the results for TGS powder, TGS + carbon powder, and TGS + Fe_3_O_4_ powder. The magnetoelectric coupling presumably appeared for the composite TGS + 10 wt. % Fe_3_O_4_, as evidenced by the shift in the phase transition temperature in the TGS. Moreover, the theoretical interpretation of the effect is proposed.

## 1. Introduction

One of the challenges of modern science is the search for new materials with multifunctional properties. In many cases, the miniaturization of electronic devices demands combining electric and magnetic properties. In this way, a single device component can perform several tasks through the synergy of multifunctional features. Magnetoelectricity (ME) implies coupling between magnetic and ferroelectric order parameters and naturally enhances the multifunctionality of the system.

Materials that exhibit magnetoelectric coupling can be divided into two groups, i.e., multiferroics and composites. In multiferroics, which are single-phase materials, ME is due to the long-range ordering of magnetic moments and electric dipoles. In turn, two types of multiferroics can be distinguished: (i) type-I, in which the temperatures of the electric ($T_{c}$) and magnetic ($T_{FM}$) transitions are higher than room temperature and $T_{c}$ > $T_{FM}$, there is a weak coupling between the ferroelectricity and magnetism, and the electric polarization has a high value (P ∼ 10 ÷ 100 μC/cm^2^); and (ii) type-II, where the transition temperatures are low and $T_{c}$≈$T_{FM}$, the coupling between the ferroelectricity and magnetism is strong, and the value of the electric polarization is small (P $∼10^{-2}$μC/cm^2^) [1]. It should be noted that not every multiferroic material is a magnetoelectric material. In composites, the ME coupling is the result of piezoelectric and magnetostrictive properties of the components. The most famous ferroelectric/piezoelectric materials are barium titanate $BaTiO_{3}$ and lead zirconate titanate (PZT) $PbZr_{x}Ti_{1-x}O_{3}$, while Terfenol-D ($Tb_{1-x}Dy_{x}Fe_{2}$ alloy) or $CoFeO_{4}$ compounds are good magnetostrictive and piezomagnetic materials, respectively.

As mentioned, in single-phase type-I multiferroics, which are very rare in nature, the ME coupling is weak and insufficient for practical use (the electric response is low at room temperature). On the other hand, in type-II multiferroics, in which the ferroelectricity is caused by a particular type of magnetism (spiral or collinear), the ME coupling may be significant; however, it occurs in the cryogenic temperature range. From the practical point of view, an easier solution is to create multiphase composite materials in which the expected magnetoelectric coupling is large and is not limited to low temperatures. As published in the literature, in particulate, multilayered, or laminated composites, the ME response of the material can be 3–5 orders of magnitude larger than in the single-phase compounds [2,3].

Research on composites of ferroelectric and ferromagnetic systems is very challenging and attractive and is in the scope of interest of numerous research groups. The term composite material refers to artificially and consciously fabricated materials that consist of at least two phases separated by distinct interfacial boundaries, where one of the phases is a reinforcing phase. A composite material (or composite) is a heterogeneous structure composed of two or more components (phases) with different properties [4,5,6,7,8,9,10].

In the present work, we focused on two materials, i.e., ferroelectric triglycine sulfate (TGS) and ferrimagnetic magnetite $Fe_{3}O_{4}$, from which the composite was made. Powdered materials with a similar level of fragmentation were combined in appropriate proportions and cold pressed, resulting in a particulate composite. We presupposed the existence of magnetoelectric coupling in the TGS + $Fe_{3}O_{4}$ composite because we observed the shift in the phase transition temperature in the experiment. The phase transition temperature in the TGS + $Fe_{3}O_{4}$ was distinct from that observed in an isolated TGS. Such an effect, i.e., a shift in the phase transition temperature caused by magnetoelectric coupling, has been observed and reported for many multiferroics [11,12,13].

Published works include separate research results for (i) TGS composites with carbon nanotubes, gelatin, polypyrrole, or nanocrystalline cellulose, or (ii) magnetite composites with polypyrrole nanotubes or zeolites. To the best of the authors’ knowledge, we produced a composite of TGS + $Fe_{3}O_{4}$ for the first time. The main objective of our study was to investigate the effect of different concentrations of magnetite doping on the dielectric properties of TGS-based composite material. Together with experimental results, we present a theoretical interpretation that, on a qualitative level, explains the experimental discovery.

## 2. Results and Discussion

As a part of this research work, a series of experiments were conducted to compare the properties of various TGS-based powder composites. Dielectric tests were conducted on the samples of a pure TGS monocrystal; powdered pure TGS; a composite based on powdered TGS and carbon powder at a concentration of 5 wt. %; and a composite based on TGS and $Fe_{3}O_{4}$ magnetite at three concentrations of 5, 10, and 20 wt. %. The powdered materials used to fabricate the composite samples had grain sizes smaller than 5 μm. Besides the dielectric studies, magnetoelectric effect measurements were made for the composites of TGS with different magnetite dopant concentrations.

### 2.1. Results of Dielectric Measurements

Dielectric measurements were carried out for four groups of samples, which were marked as follows:

- G1—monocrystalline TGS samples;
- G2—samples made of powdered TGS (grain size ≤ 5 μm);
- G3—composite samples doped with carbon powder, which is a non-magnetic material (grain size ≤ 5 μm);
- G4—composite samples doped with $Fe_{3}O_{4}$ magnetite (grain size ≤ 5 μm).

Measurements were performed using a NOVOCONTROL ALPHA, which is a dielectric spectrometer, in the frequency range from 0.01 Hz to 1 MHz and at temperatures varying from 303 to 333 K. The amplitude of the sinusoidal forcing voltage was 0.5 Vpp. For all the samples G1–G4, 3D plots of the real $\epsilon^{\prime }$ and imaginary $\epsilon^{\prime \prime }$ parts of the dielectric constant ϵ as a function of temperature and frequency were registered during the heating and cooling processes.

**Figure 1:**
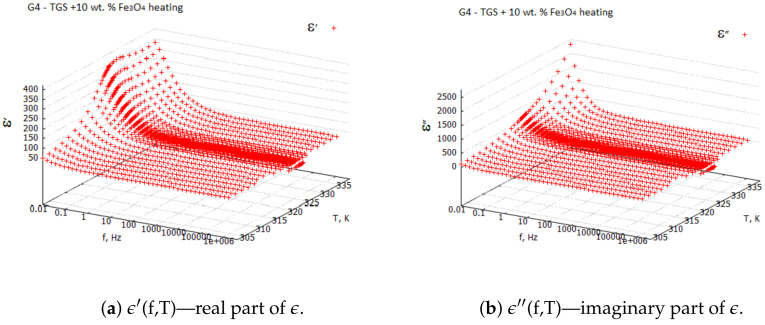
Real and imaginary components of complex dielectric constant ϵ for TGS + 10 wt. % $Fe_{3}O_{4}$—heating process.

**Figure 2:**
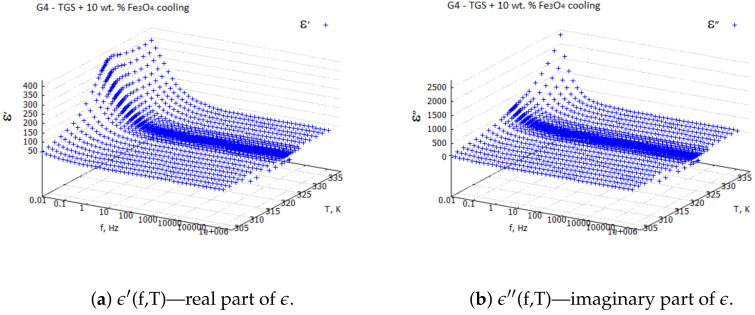
Real and imaginary components of complex dielectric constant ϵ for TGS + 10 wt. % $Fe_{3}O_{4}$—cooling process.

Examples of the 3D plots for the TGS + 10 wt. % $Fe_{3}O_{4}$ composite sample are shown in Figure 1 and Figure 2. From the 3D plots, one can choose any frequency for the observation of temperature characteristics of complex dielectric constants.

**Figure 3:**
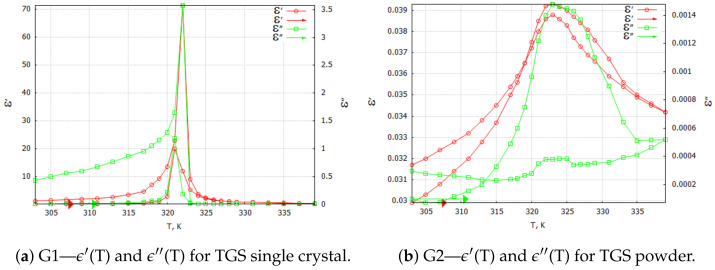
TGS single crystal and TGS powder samples: complex dielectric constant ϵ components for 1 kHz heating and cooling processes. Arrows indicate the direction of the process that was started at 303 K.

**Figure 4:**
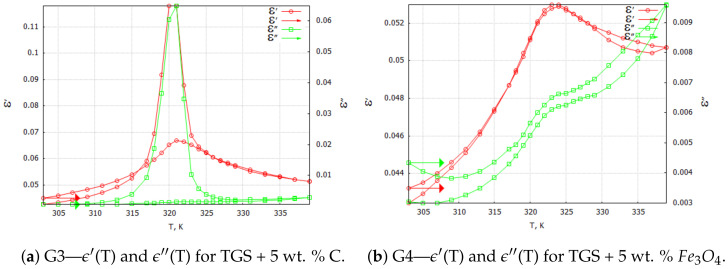
TGS powder composite samples: complex dielectric constant ϵ components for the 1 kHz heating and cooling processes. Arrows indicate the direction of the process that was started at 303 K.

**Figure 5:**
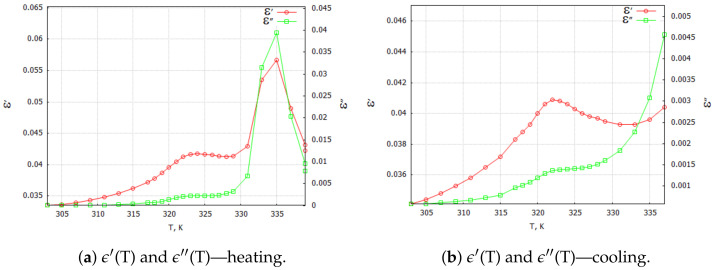
G4—TGS + 10 wt. % $Fe_{3}O_{4}$ powder composite samples: complex dielectric constant ϵ components for 1 kHz (**a**) heating and (**b**) cooling processes.

**Figure 6:**
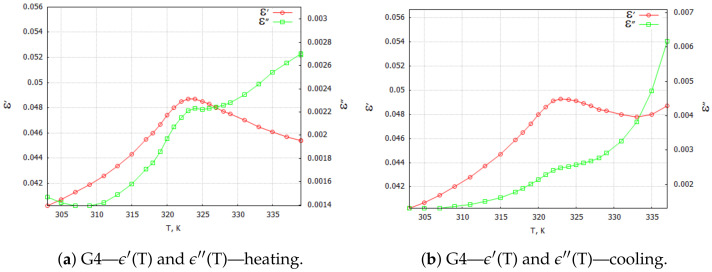
G4—TGS + 20 wt. % $Fe_{3}O_{4}$ powder composite samples: complex dielectric constant ϵ components for 1 kHz heating and cooling processes.

Three-dimensional plots of the real and imaginary parts ε’ and ε” of the complex dielectric constant $\varepsilon =\varepsilon^{\prime }+i\varepsilon^{\prime \prime }$ in the heating and cooling processes showed similarities (Figure 1 and Figure 2). In the frequency interval 0≪f≪ 1 kHz, the real parts in both cases showed plateaus corresponding to the narrow temperature interval of about ΔT=20 K. This effect had a systematic character and could be attributed to the influence of the weak ME coupling in the system. From the point of view of metrology, the measurement frequency f=1 kHz is a standard that is often used in the characterization of electronic elements. Moreover, the selection of this frequency was substantiated by the range of frequencies used in the setup for the measurements of the magnetoelectric coupling. Therefore, we examined the effect more closely and explored the case f=1 kHz with a particular interest. In Figure 3, Figure 4, Figure 5 and Figure 6, the temperature dependencies of the real and imaginary parts of the dielectric constant are plotted for the frequency f=1 kHz in the heating and then cooling processes, and for the selected samples from the groups G1–G4.

Figure 3a shows the results of the measurements for the pure TGS single-crystal sample. When focusing on the particular frequency of f=1 kHz, the real and imaginary components of the dielectric constant in the heating and cooling processes exhibited a characteristic sharp peak associated with the phase transition that occurred in the single crystal in the vicinity of the temperature $T_{c}=322$ K. The values of the complex dielectric constant components ${\epsilon^{\prime }}_{max}$ and ${\epsilon^{\prime \prime }}_{max}$ in the heating process were lower than the values in the cooling process because of depolarization of the sample during the relaxation process. This effect always preceded the measurements. The observed narrow hysteresis loop of the maximum values of the dielectric constant components can be explained by the effect of the thermal inertia of the monocrystalline sample.

Samples in the form of tablets compressed from TGS powder with a grain size < 5 μm were tested under the same conditions. In Figure 3b, a significant reduction in both the real and imaginary parts of the complex dielectric constant values can be observed. This phenomenon was due to the fragmentation and stochastic ordering of the TGS powder particles. A blurring of the peaks of the observed waveforms of $\epsilon^{\prime }$ and $\epsilon^{\prime \prime }$ was noticeable. The temperature of their onset did not change significantly. Since the powdered TGS sample did not exhibit spontaneous polarization, the aging and relaxation processes did not affect the value of the real component of the dielectric constant during the heating and cooling process. However, the powdering did affect the dielectric loss in the sample, which was evident in the waveform of the imaginary part $\epsilon^{\prime \prime }$. The powdering effectively prevented the creation of ferroelectric domains, whose presence was related to the occurrence of dielectric losses [14].

Figure 4a shows the curves of the components of the complex dielectric constant for the carbon-powder-doped TGS composite sample. Since carbon powder is a conductive material, significant differences can be observed between the heating and cooling processes. Conductive bridges that the carbon powder particles could form between dielectric grains allowed them to interact with each other. This effect was somewhat similar to the interacting areas of different polarities (ferroelectric domains in TGS single crystal). Similarly, for the case of a monocrystalline sample, one can observe characteristic sharp peaks associated with the phase transition that occurred in a TGS single crystal at about 322 K. Also, the values of the complex dielectric constant components ${\epsilon^{\prime }}_{max}$ and ${\epsilon^{\prime \prime }}_{max}$ in the heating process were lower when compared with the values in the cooling process, which was related to aging and depolarization processes.

Figure 4b illustrates the temperature dependencies of the complex dielectric constant components for the TGS + 5 wt. % $Fe_{3}O_{4}$ composite. Note that magnetite at room temperature is a dielectric. At this level of dopant content, the almost complete absence of the temperature hysteresis of $\epsilon^{\prime }$ and $\epsilon^{\prime \prime }$ could be observed. The dielectric losses were proportional to temperature, while the course of $\epsilon^{\prime }$ was almost the same as for the samples made of pure TGS powder. The range of variation of these parameters over the applied temperatures was also similar.

Special attention should be paid to the TGS + 10 wt. % $Fe_{3}O_{4}$ composite samples (Figure 5). For the samples with a 10 wt. % concentration of dopant, we observed two characteristic maxima in the temperature characteristics of $\epsilon^{\prime }$(T) and $\epsilon^{\prime \prime }$(T). The first maximum observed at about 322 K appeared to be related to the phase transition observed in the pure TGS. The second maximum, which occurrred at about 335.5 K, may have been due to the interaction between the composite phases. Traces of this interaction occurred only for the composite samples with 10 wt. % magnetite doping. One can see the differences in the shapes of the $\epsilon^{\prime }$ and $\epsilon^{\prime \prime }$ curves in the heating and cooling processes. The double peak occurred only in the heating process and only for aged samples (at least 18 h after the last heating process above $T_{c}$ = 322 K). This may mean that relaxation processes are important in the formation of interfacial interactions in the studied composite.

Figure 6 illustrates the temperature characteristics of the complex dielectric constant components in the heating and cooling processes for a composite with 20 wt. % magnetite content. The effect of the double maximum in $\epsilon^{\prime }$(T) and $\epsilon^{\prime \prime }$(T) dependencies was not observed for the tested composite with 5 wt. % (Figure 4b) and 20 wt. % (Figure 6) magnetite doping concentrations.

We believe that the occurrence of a blurring and shifting of the characteristic point of the phase transition (Figure 5a) compared with the pure TGS powder may indicate the presence of an interaction between the magnetic (M) and electric (E) phases in the composite material, i.e., ME coupling in the system. Such a phenomenon is discussed in the theoretical model presented in the next section. The results of the experiments that were necessary to fit the theoretical model were determined from the 3D plots of the real and imaginary components of the dielectric constant and are listed in Table 1 for the composite TGS + 10 wt. % $Fe_{3}O_{4}$. The parameters are presented for the lowest frequency (0.01 Hz—marked as static); for the highest frequency (1 MHz—marked as infinity); and for three different temperatures: 303, 331 and 338 K.

### 2.2. Results of Magnetoelectric Coupling Measurements

Since the shift of the phase transition temperature was observed only for the composite sample G4—TGS + 10 wt. % $Fe_{3}O_{4}$, special attention was paid to the measurements of the magnetoelectric coupling for this group of samples. We used the dynamic method (described in detail in the next section). In this technique, the sample is placed in two mutually parallel magnetic fields, i.e., a constant $H_{DC}$ and variable $H_{AC}$, and the voltage signal is measured on electrodes on the edges of the sample. At a selected frequency and intensity of an alternating magnetic field, the dependence of the sample voltage U on the constant magnetic field bias is examined. A voltage signal should appear on the sample electrodes if the material exhibits magnetoelectric coupling. In our study, for an alternating magnetic field frequency of f=1 kHz and $H_{AC}$ = 4 Oe, we observed weak voltage signals for the G4—TGS + 10 wt. % $Fe_{3}O_{4}$ samples.

**Figure 7:**
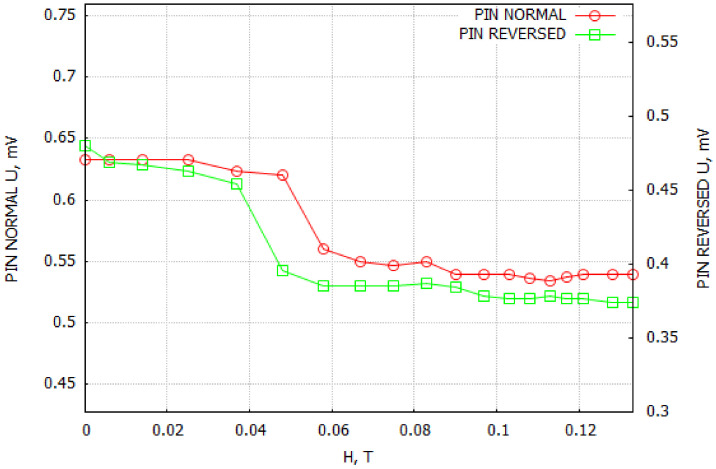
ME coupling measurements. Pin normal—the holder was an element of the BNC plug, pin reversed—the sample was turned 180°.

As seen in Figure 7, the recorded voltage varied over a small range. In order to eliminate the influence of sample mounting, measurements were performed in two variants (Figure 7: pin normal—the holder was an element of the BNC plug, pin reversed—the sample was turned 180°). The observed slight slope of the measured voltage at 0.05 T could be connected to the noise interference from the experimental setup.

Many authors observed the ME effect in multiferroic materials through the shift of the phase transition temperature [15,16,17]. The measurements made by us in both the presented methods confirmed the existence of the ME coupling in the composite sample G4—TGS + 10 wt. % $Fe_{3}O_{4}$. However, such an effect was weak because the mechanical contact between the TGS and $Fe_{3}O_{4}$ grains was not strong enough to induce a substantial screening effect of the electric charge near the interface. Furthermore, the screening effect was not strong enough to induce a substantial magnetostriction response of the magnetite dopant and excite a significant piezoelectric effect in the TGS.

## 3. Materials and Methods

### 3.1. Magnetic Part of the Composite

Magnetite, with the chemical formula of $Fe_{3}O_{4}$, is a ferrimagnetic material. It demonstrates an inverse-spinel cubic structure and belongs to one of the essential compounds in materials engineering. It has a relatively high saturation magnetization in the order of 98 emu/g [18] and a low coercive field value of 300 Oe at 353 K [19]. The Curie temperature is about 773 K. The material is widely used in numerous applications, such as the biosensors industry, automotive industry, and medicine (drug delivery, MRI contrast, etc. [20]). In general, $Fe_{3}O_{4}$ is a good absorbent for electromagnetic waves, making this material perfect for radar-absorbing applications (absorbing microwaves, dampening reflections). The absorption surface removes the electric or magnetic field from the wave and transforms it into heat [21]. Magnetic order in ferrimagnetic materials is formed by magnetic moments of atoms of different sublattices with an antiparallel configuration below the critical temperature [22,23]. While magnetic order is antiferromagnetic, magnetic moments in sublattices are different and do not compensate for each other, leading to net spontaneous magnetization without an external magnetic field. Magnetite is a perfect example of a ferrimagnetic material with significant temperature-dependent magnetic susceptibility. When using magnetite in the synthesized composites, it should be noted that the magnetic properties of ferrimagnetic materials may vary depending on the grain size [24]. For the experiments, we used commercially available magnetite (SIGMA-ALDRICH Iron II, III oxide powder 5 μm).

### 3.2. Electric Part of the Composite

Triglycine sulfate (TGS), with the chemical formula ${\left(NH_{2}CH_{2}COOH\right)}_{3}H_{2}SO_{4}$, is a hybrid organic–inorganic material that is relatively easily obtained in the form of single crystals [25]. It is one of the most comprehensively studied ferroelectric materials for infrared, non-cooled thermal detectors (FTIR spectroscopy, thermal imaging, night vision, remote temperature sensing, etc.). Unlike quantum detectors, TGS-based detectors are uniformly sensitive to radiation over a very wide range of wavelengths, from the ultraviolet to the far infrared. The mechanism of the phase transition in TGS single crystals and its electric properties has been the subject of numerous scientific papers [26,27,28,29,30,31]. Studies have been carried out on the doping of pure TGS with organic and inorganic compounds [32,33,34]. Many authors have studied the properties of thin films and composite materials using TGS [35,36,37,38,39,40]. The influence of a magnetic field on the electrical properties of monocrystalline TGS samples has also been studied [41].

TGS is a hydrogen-bonded ferroelectric crystal with a typical second-order phase transition at a Curie temperature of about 322 K. It is a paradigmatic model of a uniaxial ferroelectric and is attractive because of its excellent pyroelectric properties, high figures of merit, and easy growth process. During the phase transition, the crystal symmetry changes from non-ferroelectric $P_{21/m}$ in the high-temperature phase to ferroelectric $P_{21}$ below $T_{C}$. For our experiments, pure single crystals of TGS were grown from a water solution of stoichiometric quantities of amino-acetic and sulfuric acids using a static method of water evaporation [42]. Appropriate amounts of amino-acetic acid and sulfuric acid were dissolved in demineralized high-purity water (electrical conductivity 0.05 μS/cm) obtained with the use of a demineralization and reverse osmosis system Polwater Deionizer DL2-100 (Polwater, Krakow, Poland). The following equation describes the chemical reaction for the formation of triglycine sulfate:

(1) $$\begin{array}{c} 3\left(NH_{2}CH_{2}COOH\right)+H_{2}SO_{4}={\left(NH_{2}CH_{2}COOH\right)}_{3}\times H_{2}SO_{4} \end{array}$$

**Figure 8:**
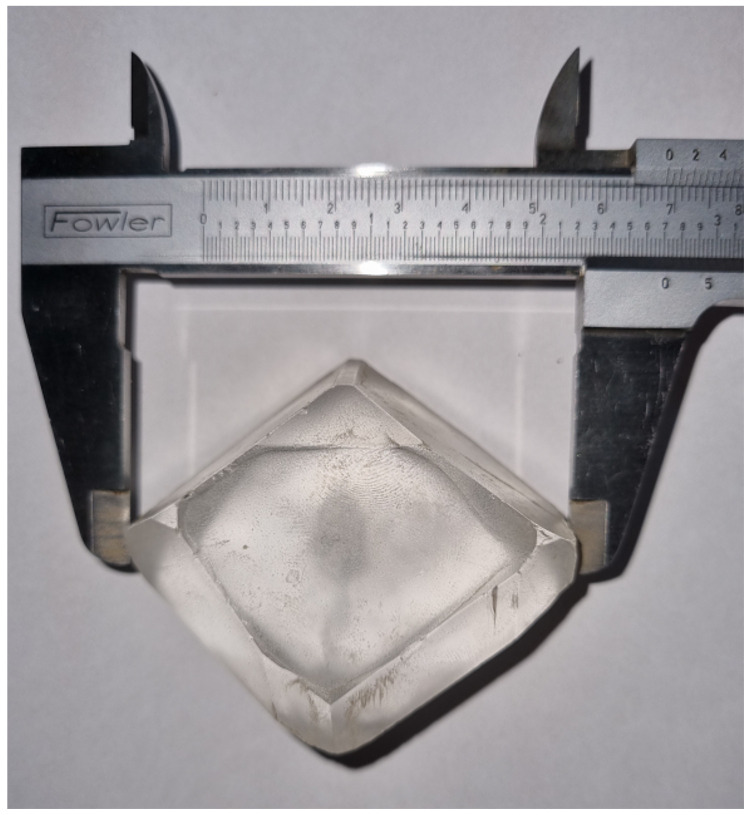
Example of grown TGS single crystal.

Such an obtained solution was left to crystallize. When the spontaneous crystallization occurred at the bottom of the crystallization vessel, tiny single crystals were taken off and treated as the seeds for regular crystal growth. The seeds obtained in this way were mounted on the end of a rotative handle and plunged into the solution of TGS. The handle was rotated at a speed of 40 rpm. The evaporation speed was experimentally adjusted by changing the size of the ventilation slots in the lid of the crystallization vessel. TGS single crystals used for the experiment were grown in the paraelectric phase at a temperature of 323 K. Figure 8 shows a photo of one of the largest TGS monocrystals we grew in our lab. Usually, grown monocrystals do not exceed about 3 cm in their longest dimension.

The mechanism of the structural phase transition in TGS can be explained by two structural changes when crossing the $T_{c}$ temperature. The first one is related to the reorientation of the $-NH_{3}^{+}$ group of glycine $G_{I}$ (a statistically averaged mirror position above $T_{c}$). The second one relates to the disordering of the H bond between two glycines $G_{II}$ and $G_{III}$. These mechanisms make the two glycine ions indistinguishable at temperatures above $T_{c}$. The molecular motions mentioned above are coupled through the hydrogen-bonding network of the crystal. This process can be influenced by external factors, such as the pressure or isotope substitution of hydrogen atoms by deuteron at the single-crystal growth stage [43]. Since the interaction of the external magnetic field with TGS monocrystalline samples was experimentally confirmed [41,44], it was decided to check the effect of a magnetic dopant on the TGS-based powder composite.

### 3.3. Composite Fabrication

**Figure 9:**
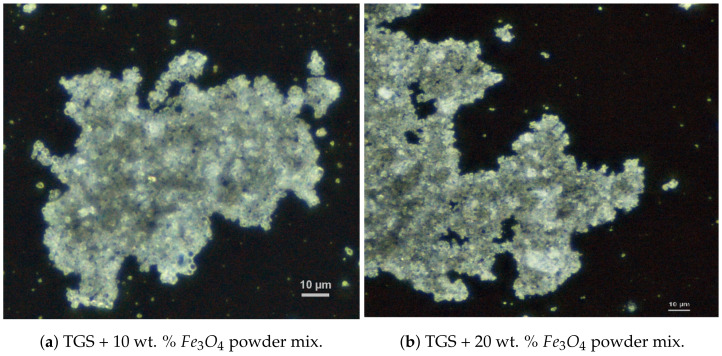
Images of the grain structure of the composite with different dopant contents.

To prepare the composite, we used commercially available $Fe_{3}O_{4}$ powder and powdered TGS single crystals obtained in our laboratory. The grain diameters of both materials were confirmed using the NICON Clips LV 100 POL polarizing microscope. Examples of images of powdered TGS doped with two different concentrations of magnetite (10 and 20 wt. %) are shown in Figure 9.

In order to obtain powdered TGS with the assumed granulation (5 μm), the single crystals from the growing process were pre-broken in a mortar and then ground in a ball mill. A zirconium bowl and zirconium balls with a diameter of 10 mm were used for the grinding. In order to avoid clumping of the ground mass, the process was carried out in the presence of liquid isopropanol IPA 99.9% (IMAX).

The process of preparation of the composite had three stages. Stage 1: After the initial breaking of the single crystals in a mortar, the TGS grinding process in a ball mill was carried out at a speed of 130 rpm in several 10-min cycles. At the end of each cycle, a sample of the resulting powder was taken to check the grain gradation. Stage 2: After obtaining a gradation of about 5 μm, the obtained powder portion was divided into two parts. About 1/3 of the portion was used to produce samples without admixtures. The remaining portion was used to create composite samples with various dopants, i.e., carbon and magnetite with 5, 10, and 20 wt. % concentrations. Stage 3: The powder obtained by dry grinding in the previous step was used to prepare a paste from which the final samples were extruded. Isopropyl alcohol was added to the TGS powder, and, in the case of creating doped samples, the proper weight percentage amount of the dopant was added at the same time. Appropriate amounts of powder were weighed using a RADWAG AS220 RZ PLUS laboratory scale (Radwag, Radom, Poland). The grinding was carried out while wet at a speed of 850 rpm in thirty 1 min cycles. Successive cycles took place with a change in the direction of rotation of the mill head.

The resulting composite paste was cold-pressed in an LFA VICE Handheld Pill tablet press. The applied pressure in the case of all samples was not less than 20 kN. The samples were then dried at 303 K for 24 h. Conductive silver electrodes were applied to the samples after drying. The diameter of the pressed samples was $8\times 10^{-3}$m and their thickness varied between $2\times 10^{-3}$ and $2.5\times 10^{-3}$ m.

Reference mono-crystalline samples were cut perpendicular to the ferroelectric b-axis from oriented TGS single crystals of good optical quality grown as described in the previous subsection. After the mechanical treatment, samples with dimensions of about $1\times 10^{-2}$ m (width), $2\times 10^{-2}$ m (length), and $2\times 10^{-3}$ m (thickness) were formed. Silver electrodes were attached to the plates of such formed samples.

### 3.4. Measurements Methods

**Figure 10:**
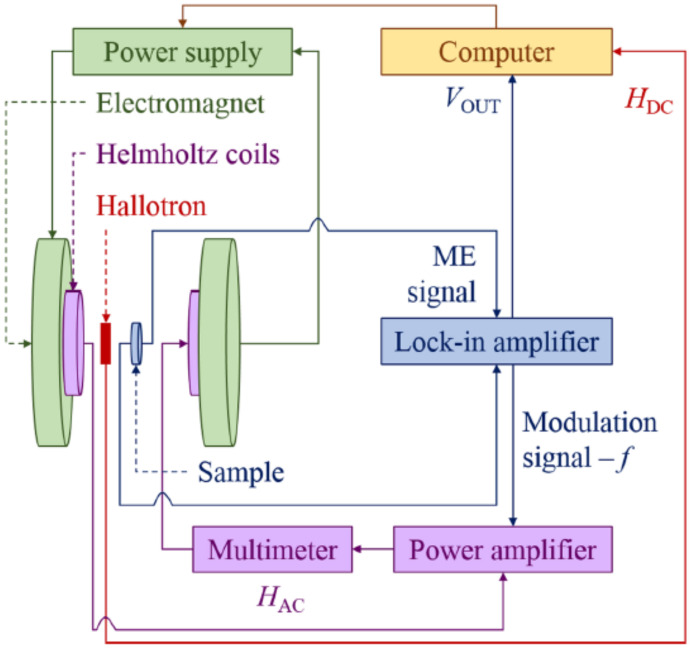
The block diagram of ME coupling effect measuring system [45].

Electric properties of the samples (e.g., dielectric permittivity, electrical capacity, and loss angle tangent) were examined with the use of the NOVOCONTROL ALPHA-A dielectric spectrometer (Novocontrol, Montabaur, Germany) and AGILENT HP 4991 impedance analyzer (Agilent, Waldbronn, Germany). Measurements of complex dielectric constant components used for the mathematical model were carried out in 24 h cycles, meaning that the samples had at least 18 h of aging, during which the relaxation processes could take place. In the experiment, we used a frequency range from 0.01 Hz to 1 MHz and temperature between 303 and 338 K. For the measurements of ME coupling, we used our homemade system with the dynamic lock-in technique described in detail in [45,46]. The block diagram of the ME coupling effect measuring system is presented in Figure 10.

A sample, usually in the form of a flat disc with electrodes, was placed inside a pair of Helmholtz coils, which were positioned between the poles of an electromagnet. The coils generated a uniform AC magnetic field that was superimposed over a DC magnetic field produced by the electromagnet. Both AC and DC field lines ran parallel to each other and were perpendicular to the sample surface. The magnetic flux density near the sample was measured with a Hall probe. The changing magnetic field $H_{AC}$ induced a magnetoelectric voltage across the sample. The voltage signal was then processed by the frequency-attuned lock-in amplifier (SR830 DSP). The measurements were performed at room temperature for the alternating magnetic field frequency of f = 1 kHz and $H_{AC}$ = 4 Oe. To generate the DC magnetic field, an electromagnet with a fixed gap was employed, and the induction of the constant magnetic field was changed between 0 and 0.13 T.

This kind of measurement is essential in applications related to materials engineering because the exploitation of the ME materials in electronic devices requires both the setting of the operating point by selecting the $H_{DC}$ and the usable signal at a specific frequency *f* at which the magnetoelectric coupling will be the most effective.

## 4. Theoretical Model

In what follows, we provide a comprehensible theoretical explanation of the effects observed in the experiment. The dynamics of the ferroelectric polarization at finite temperatures is the essence of the stochastic process described by the Ginzburg–Landau equation [47]:

(2) $$\begin{array}{c} \frac{\partial P(r,t)}{\partial t}=-\Gamma \frac{\delta F}{\delta P(r,t)}+\xi \left(r,t\right). \end{array}$$

Here, ∂P(r,t) is the time-dependent ferroelectric polarization density; Γ is the dissipation parameter; and ξ(r,t) is the stochastic noise related to the effect of the finite temperature and characterized by the correlation function $\langle \xi \left(r^{\prime },t^{\prime }\right)\xi \left(r,t\right)\rangle =\sigma^{2}\delta \left(r^{\prime }-r\right)\delta \left(t^{\prime }-t\right)$, where $\sigma^{2}=\Gamma \left(k_{B}T/V\right)$ is the effective temperature quantifying the noise that depends on the characteristic volume of the ferroelectric sample *V*. For the sake of simplicity in the theoretical discussion, we adopt dimensionless polarization *P* and magnetization *M* order parameters. The free energy of the ferroelectric system has the form:

(3) $$F\left(\gamma \right)=aP^{2}+bP^{4}-EP-\gamma P^{2}M^{2},$$

where $a=-A(T_{c}-T)$ and $T_{c}$ is the temperature of the phase transition. The first two terms in Equation (3) describe the biharmonic $\varphi^{4}$ double-well potential; the third term describes the interaction of the ferroelectric system with the external electric field *EP*; and the last term, i.e., $\gamma P^{2}M^{2}$, describes the the magnetoelectric effect coupling between ferroelectric and ferromagnetic order parameters. We assumed that the system was uniform, and we neglected spatial dependence. We implemented the Kramers–Moyal method [48] and derived the Fokker–Planck (FP) equation for the ferroelectric system [49]:

(4) $$\begin{array}{c} W\left(P,t\right)={\hat{L}}_{FP}W\left(P,t\right),\\ L_{FP}=-\frac{\partial }{\partial P}D^{\left(1\right)}+\frac{\partial^{2}}{\partial P^{2}}D^{\left(2\right)}. \end{array}$$

Here, the Kramers–Moyal coefficients are given by

(5) $$D^{\left(n\right)}=\frac{1}{n!}\lim_{\left\{\tau \to 0\right\}}\frac{1}{\tau }\langle {\left[P\left(t+\tau \right)-P\right]}^{n}\rangle {|}_{P(t)=P},$$

P(t+τ) is the solution of Equation (2), which at time *t* has the sharp value P(t)=P, and the function W(P,t) quantifies the probability distribution of the ferroelectric polarization. The explicit forms of the coefficients read $D^{\left(1\right)}=-2a\Gamma P-4b\Gamma P^{3}+\Gamma E+2\gamma \Gamma M^{2}P$ and $D^{\left(2\right)}=2Dk_{B}T/\left(V\Gamma \right)$. Solving the time-dependent FP equation is a mathematically demanding problem. We implemented the supersymmetric method [49]. We present the solution of the FP equation in the following form:

(6) $$\begin{array}{c} W\left(P,t\right)=\psi \left(P,t\right)\exp\left[-\frac{\beta }{2}F\left(P\right)\right], \end{array}$$

where $\beta =1/\sigma^{2}$ is the effective inverse temperature and ψ(P,t) is the solution of the Schrödinger equation for the imaginary time:

(7) $$\begin{array}{c} \frac{\partial \psi }{\partial t}=-\hat{H}\psi \left(P,t\right), \end{array}$$

with the following Hamiltonian:

(8) $$\begin{array}{c} \hat{H}=-\frac{d^{2}}{dP^{2}}+{\left(\frac{F^{\prime }\left(P\right)}{2\sigma^{2}}\right)}^{2}-\frac{F^{\prime \prime }\left(P\right)}{2\sigma^{2}}. \end{array}$$

By equating the stationary parts of the solution of the Fokker–Planck equation, we estimated the shift of the effective temperature in the system associated with the ME term $\Delta T=T_{\gamma }-T_{0}=-\frac{\gamma p^{2}M^{2}}{F(\gamma =0)}T_{0}$. Here, $T_{0}$ and $F_{0}$ are the temperature and energy of the ferroelectric system in the absence of the ME coupling. The obtained result means that the temperature $T_{0}$ in the ferroelectric system decoupled from the magnetic layer is equivalent to the temperature $T_{0}-\frac{\gamma p^{2}M^{2}}{F(\gamma =0)}T_{0}$ in the presence of the ME coupling. To estimate the shift of the phase transition temperature, we find the roots of the following equation:

(9) $$\begin{array}{c} \frac{dF(p)}{dp}=-2\left[A\left(T_{c}-T\right)+\gamma M^{2}\right]p+4bp^{3}-E=0. \end{array}$$

The minimum of the energy corresponds to

(10) $$p_{0}=\frac{2}{\sqrt{3}}\sqrt{\frac{A\left(T_{c}-T\right)+\gamma M^{2}}{2b}}\cos\theta /3,$$

Here, $\theta =\arccos\left(\frac{3E\sqrt{b}}{{\left(A\left(T_{c}-T\right)+\gamma M^{2}\right)}^{3/2}}\right)$ and $T_{c}$ is the phase transition temperature in the absence of the ME coupling. As we can see, Equation (10) contains several parameters, such as the phenomenological constants *A* and *b* and the ME coupling constant γ. Therefore, we can eliminate two unknown parameters in future experiments by performing two independent measurements of the polarization and steady-state magnetization at different temperatures *T*. The polarization is still finite at $T_{c}$, and the phase transition occurs at $T_{c}^{\prime }=T_{c}-\frac{\gamma p^{2}M^{2}}{F(\gamma =0)}T_{c}$. This last equation defines the shift of the phase transition temperature. In our experiment (see Figure 3b and Figure 5a): $T_{c}=336$ K, $T_{c}^{\prime }=322$ K, ΔT=−14 K, and $\Delta F\left(\gamma \right)=F\left(\gamma \right)-F\left(0\right)=-\gamma p_{0}^{2}M^{2}=0.04F\left(0\right)$, where F(0)=F(γ=0).

To estimate relaxation time, we solve the following eigenvalue problem:

(11) $$\begin{array}{c} \hat{H}\psi_{n}=\lambda_{n}\psi_{n}. \end{array}$$

In the limit of zero electric field E=0, we neglect the higher-order polynomial term proportional to $p_{0}^{4}$. The supersymmetric bosonic potential has the following form [49]:

(12) $$V_{+}={\left[\frac{1}{2}\beta F^{\prime }\left(p\right)\right]}^{2}+\frac{1}{2}\beta F^{\prime \prime }\left(p\right).$$

Taking into account the form of the bosonic potential for relaxation time, we deduce $\tau_{FP}=1/\lambda_{2}$:

(13) $$\tau_{FP}=\left(\frac{\hbar }{F(p_{0})}\right)\frac{k_{B}T\Gamma }{\left[p_{0}^{2}\left(A\left(T_{c}-T\right)+\gamma M^{2}\right)\right]}.$$

As we can see, a negative ME coupling constant γ<0 increases the barrier and increases the relaxation time. The relaxation time defines the formation of the steady phase in the system when observing the thermal time transition process in the time domain. Following the phenomenological Havriliak–Negami equation [47], the symmetric Cole–Cole function reads

(14) $$\varepsilon^{\prime }-i\varepsilon^{\prime \prime }=\epsilon_{\infty }+\frac{\Delta \varepsilon }{\left[1+{\left(i\omega \tau \right)}^{1-\alpha }\right]}.$$

Here, 0⩽α⩽1 is the width parameter, Δε is the relaxation strength, and $\varepsilon_{\infty }$ is the high-frequency limit of the dielectric constant. For simplicity, we take α=0; then, we deduce

(15) $$\begin{array}{c} \varepsilon^{\prime }=\epsilon_{\infty }+\frac{\Delta \varepsilon }{1+\omega^{2}\tau^{2}},\\ \varepsilon^{\prime \prime }=\frac{\omega \tau \Delta \varepsilon }{1+\omega^{2}\tau^{2}}, \end{array}$$

or in the equivalent form:

(16) $$\begin{array}{c} \Delta \varepsilon =\left(\varepsilon^{\prime }-\epsilon_{\infty }\right)\left(1+\omega^{2}\tau^{2}\right),\\ \tau =\frac{\varepsilon^{\prime \prime }}{\omega (\varepsilon^{\prime }-\epsilon_{\infty })}. \end{array}$$

Taking into account the experimental values of $\varepsilon^{\prime }=350$, $\varepsilon^{\prime \prime }=800$, ω = 1 MHz, and $\epsilon_{\infty }=21$, we find $\tau =2.3\times 10^{-6}$ s. The experimental value of τ can be compared with the theoretical estimation from Equation (13). However, for a quantitative analysis, one needs to extract the parameters *A*, *M*, and *b* from further experiments. The proposed theoretical model qualitatively explains the central experimental founding at the current stage: the shift of the phase transition temperature due to the ME coupling.

## 5. Conclusions

This study dealt with composite materials and, more specifically, with the creation of new materials with potential applications in the electronics industry. Within our best knowledge, we can expect some applications of the composite as a material for sensors or absorption/reflection layers. At the preliminary stage, a selection of materials with promising properties was made. Two flagship materials were selected: TGS from the group of electric materials (E) and magnetite from the group of magnetic materials (M). To our best knowledge, no attempt was previously made to study the properties of particulate composites using these two materials. As a part of the completed research work, TGS + $Fe_{3}O_{4}$ composite samples with three magnetite doping contents (5, 10, 20 wt. %) were produced and tested. The study of the dielectric properties of the composite was carried out using broadband dielectric spectroscopy methods. The study of ME coupling was performed using a homemade system that implemented the dynamic lock-in technique. For the composite samples with 10 wt. % magnetite content, it was found that there was a 14 K shift in the phase transition point when compared with the reference samples of pure TGS powder. Such a phenomenon has been reported in numerous publications for other composite materials. The observed effect may indicate the existence of ME coupling in the studied composite. In the theoretical part, it was confirmed that the interfacial ME interaction in the composite may lead to a change in the dynamic parameters of the material (relaxation time) and cause a shift in the phase transition point with respect to the constituent materials as a reference.

The magnetoelectric effect observed in our experiment was small compared with the theoretically predicted ferroelectric system’s characteristic free energy. The paper contains the first preliminary results on a TGS-based multiferroic composite obtained by a cold method. This topic requires further research and will be the subject of our future research work.

## Figures and Tables

**Table 1:** Critical values of dielectric constant at selected temperatures measured for TGS + 10 wt. % $Fe_{3}O_{4}$ sample during heating and cooling processes.

Heating	ϵ’	ϵ”
303 K static	48	16
303 K infinity	90	0.3
326 K static	350	800
326 K infinity	21	0.2
338 K static	330	2300
338 K infinity	20	0.5
**Cooling**	** $\epsilon^{\prime }$ **	** $\epsilon^{\prime \prime }$ **
338 K static	330	2300
338 K infinity	20	1.5
326 K static	360	760
326 K infinity	22	0.3
303 K static	48	75
303 K infinity	17	0.3

## Data Availability

Data are contained within the article.
